# JAK inhibitors disrupt T cell-induced proinflammatory macrophage activation

**DOI:** 10.1136/rmdopen-2022-002671

**Published:** 2023-01-04

**Authors:** Mukanthu H Nyirenda, Jagtar Singh Nijjar, Marina Frleta-Gilchrist, Derek S Gilchrist, Duncan Porter, Stefan Siebert, Carl S Goodyear, Iain B McInnes

**Affiliations:** 1 School of Infection and Immunity, University of Glasgow College of Medical Veterinary and Life Sciences, Glasgow, UK; 2 The Research into inflammatory Arthritis Centre of Excellence - Versus Arthritis (RACE-VA) Universities of Glasgow, Birmingham, Newcastle, Oxford, and Newcastle, UK; 3 Gartnavel General Hospital, Glasgow, UK; 4 College of Medical Veterinary and Life Sciences, University of Glasgow, Glasgow, UK

**Keywords:** antirheumatic agents, arthritis, rheumatoid, chemokines, inflammation, T-lymphocyte subsets

## Abstract

**Objectives:**

Macrophage subsets, activated by T cells, are increasingly recognised to play a central role in rheumatoid arthritis (RA) pathogenesis. Janus kinase (JAK) inhibitors have proven beneficial clinical effects in RA. In this study, we investigated the effect of JAK inhibitors on the generation of cytokine-activated T (Tck) cells and the production of cytokines and chemokines induced by Tck cell/macrophage interactions.

**Methods:**

CD14^+^ monocytes and CD4^+^ T cells were purified from peripheral blood mononuclear cells from buffy coats of healthy donors. As representative JAK inhibitors, tofacitinib or ruxolitinib were added during Tck cell differentiation. Previously validated protocols were used to generate macrophages and Tck cells from monocytes and CD4^+^ T cells, respectively. Cytokine and chemokine including TNF, IL-6, IL-15, IL-RA, IL-10, MIP1α, MIP1β and IP10 were measured by ELISA.

**Results:**

JAK inhibitors prevented cytokine-induced maturation of Tck cells and decreased the production of proinflammatory cytokines TNF, IL-6, IL-15, IL-1RA and the chemokines IL-10, MIP1α, MIP1β, IP10 by Tck cell-activated macrophages in vitro (p<0.05).

**Conclusions:**

Our findings show that JAK inhibition disrupts T cell-induced macrophage activation and reduces downstream proinflammatory cytokine and chemokine responses, suggesting that suppressing the T cell-macrophage interaction contributes to the therapeutic effect of JAK inhibitors.

**WHAT IS ALREADY KNOWN ON THIS TOPIC**

- Janus kinase (JAK) inhibitors modulate the activity of proinflammatory cytokines implicated in rheumatoid arthritis (RA progression.

**WHAT THIS STUDY ADDS**

- JAK inhibition disrupts cytokine-activated T cell-induced macrophage activation, an in vitro model for RA synovial T-cell-mediated stimulation of monocytes/macrophages, and reduces proinflammatory cytokine and chemokine responses.
- Suppression of the T cell: macrophage interaction may contribute to the therapeutic efficacy of JAK inhibitors.

**HOW THIS STUDY MIGHT AFFECT RESEARCH, PRACTICE OR POLICY**

- Although this study may not directly change policy or practice, it highlights that JAK inhibition may affect multiple steps involved in mediating inflammation by inhibiting cytokine and chemokine production and disrupting the function of innate and acquired immune cells.

## Introduction

The hallmarks of synovitis in rheumatoid arthritis (RA) include the infiltration of inflammatory cells, predominantly blood-derived lymphoid and myeloid cells.^1^ Infiltrating T-cells and monocytes/macrophages reside collaboratively within the inflamed RA synovium; hence, the established heterogeneity of inflammatory synovial macrophages^2^ can drive synovitis via cytokine production by activated T-cells.^3^ Conversely, T-cells activate macrophages, leading to a proinflammatory cytokine forward feedback loop.^4^

Perpetuation of inflammation by monocytes/macrophages stimulated by cytokine-activated T (Tck) cells may be modelled in vitro by the macrophage: T-cell coculture assay.^5 6^ Signalling pathways elicited by Tck cells in responding monocytes were identical to those induced by T-cells isolated from RA synovial tissue.^6 7^ Thus, Tck cells have been proposed as a model for RA synovial T-cell-mediated stimulation of monocytes/macrophages via cell-cell contact-dependent mechanisms.^5 7^

Janus kinases (JAKs) are tyrosine kinases that recruit and activate signal transducers and activators of transcription, which drive proinflammatory cellular responses. There are four JAKs in humans, JAK1, JAK2, JAK3 and tyrosine kinase 2 (TYK2)^8^ that subserve the biology of a range of cytokines critical in the pathogenesis of RA.^9^ The JAK inhibitor tofacitinib interacts with multiple JAKs, although preferentially inhibiting JAK1-dependent and JAK3-dependent cytokines.^10^ At the pathophysiological level, tofacitinib and now other JAK inhibitors, modulate the activity of proinflammatory cytokines implicated in RA progression.^10 11^ Though the biological roles of JAK inhibitors in lymphocytes are well known, their function in the context of macrophage: T-cell interactions has not been defined. Here, we investigated the effect of tofacitinib, ruxolitinib and Tyrphostin AG-490 on the generation of Tck cells and assessed whether JAK inhibition in Tck cells impacts cytokine and chemokine production by macrophages in the vitro coculture system.

## Materials and methods

### Isolation of peripheral blood mononuclear cells from buffy coats

Peripheral blood mononuclear cells (PBMC) were isolated from buffy coats using Histopaque 1077 (Sigma) density centrifugation. The buffy coats were obtained from healthy volunteers from the Scottish National Blood Transfusion Service UK (SNBTS). All subjects provided informed consent with the appropriate ethical approvals in place.

### Reagents

Tofacitinib, ruxolitinib and Tyrphostin AG-490 were obtained from LC-Labs and dissolved in dimethyl sulfoxide (DMSO) (Sigma). Serial dilutions were employed to ensure that DMSO concentration did not exceed 0.001% in cell culture. Recombinant TNF, IL-6, IL-2 and MCSF (Peprotech); IFN-γ, GM-CSF and IL-4 (R&D Systems); Lipopolysaccharide (Salmonella typhimurium; Sigma).

### Purification of CD14^+^ monocytes and differentiation into macrophages

CD14^+^ monocytes were purified by positive magnetic separation using CD14 immunomagnetic microbeads (Miltenyi Biotec). Purities of >98% were routinely confirmed by flow cytometry (data not shown). CD14^+^ monocytes were cultured in 24 well-plates with MCSF (50 ng/mL) for 3 days. Cells were detached using a non-enzymatic dissociation solution (Sigma), washed in Dulbecco’s phosphate-buffered saline and then replated in 96 well plates at a density of 5×10^5^ cells/mL with MCSF (50 ng/mL) for the additional 3 days before harvesting and used in subsequent experiments.

### Purification and differentiation of CD4^+^ Tck cells and the effect of JAK inhibition on Tck cell maturation

CD4^+^ T-cells were isolated from PBMC using CD4 microbeads (Miltenyi Biotec). To generate Tck cells, CD4^+^ T-cells were cultured at 1×10^6^ cells/mL with IL-2 (25 ng/mL), IL-6 (100 ng/mL) and TNF (25 ng/mL) (Peprotech) for 6 days.^12^ In separate experiments, the JAK inhibitor tofacitinib was added to the culture medium during the 6-day maturation period to assess the effect of tofacitinib on Tck cell maturation. The cells were washed and cultured with mature macrophages at a 4:1 Tck: macrophage ratio. The supernatants were collected and assessed for TNF using ELISA.

### Macrophage activation by Tck cells in coculture and the effect of JAK inhibition

Fixed numbers of monocyte-derived macrophages (5.0×10^5^ cells) were cocultured with autologous Tck cells, achieving macrophage: Tck ratios of 1:8, 1:4, 1:2 and 1:1. Macrophages and Tck cells were also cultured alone. A 1:4 macrophage/Tck cell ratio was optimal and was used to assess the effect of JAK inhibitors. DMSO (0.001%) was used as a control. The cocultured cells were incubated for 24 hours, and supernatants were collected for analysis using ELISA or Luminex. Trans-well inserts (0.4 µm pore size; Corning) were used to assess cell-cell contact independent mechanisms.

### Measurement of cytokines in culture supernatants

IL-6, IL-15, IL-1RA, IL-10, MIP1α, MIP1β, IP10 and MIG were assessed using Luminex (Invitrogen), and TNF was determined using ELISA (Invitrogen); all following the manufacturer’s protocols.

### Statistical analysis

Results were analysed using GraphPad Prism V.9 Software, (San Diego, California, USA). Statistical differences between groups were analysed using the Wilcoxon rank test, Student’s t-test or one-way analysis of variance with Bonferroni post hoc corrections as appropriate.

## Results

### Tck induce contact-dependent and concentration-dependent TNF production by human macrophages

To delineate the effect of JAK inhibition on macrophages, we revisited the generation of Tck cells and their ability to activate monocyte-derived macrophages. In coculture, we established a 1:4 macrophage/Tck cell ratio as the optimal ratio of macrophages to Tck cells. In agreement with previous reports,^12 13^ Tck cells activated macrophages contact-dependently, secreting TNF (online supplemental figure 1A, B).

10.1136/rmdopen-2022-002671.supp1 Supplementary data

### JAK inhibitors prevent cytokine-induced maturation of Tck cells and inhibit the production of IL-6, IL-15 and IL-1RA by TcK-activated macrophages

**Figure 1:**
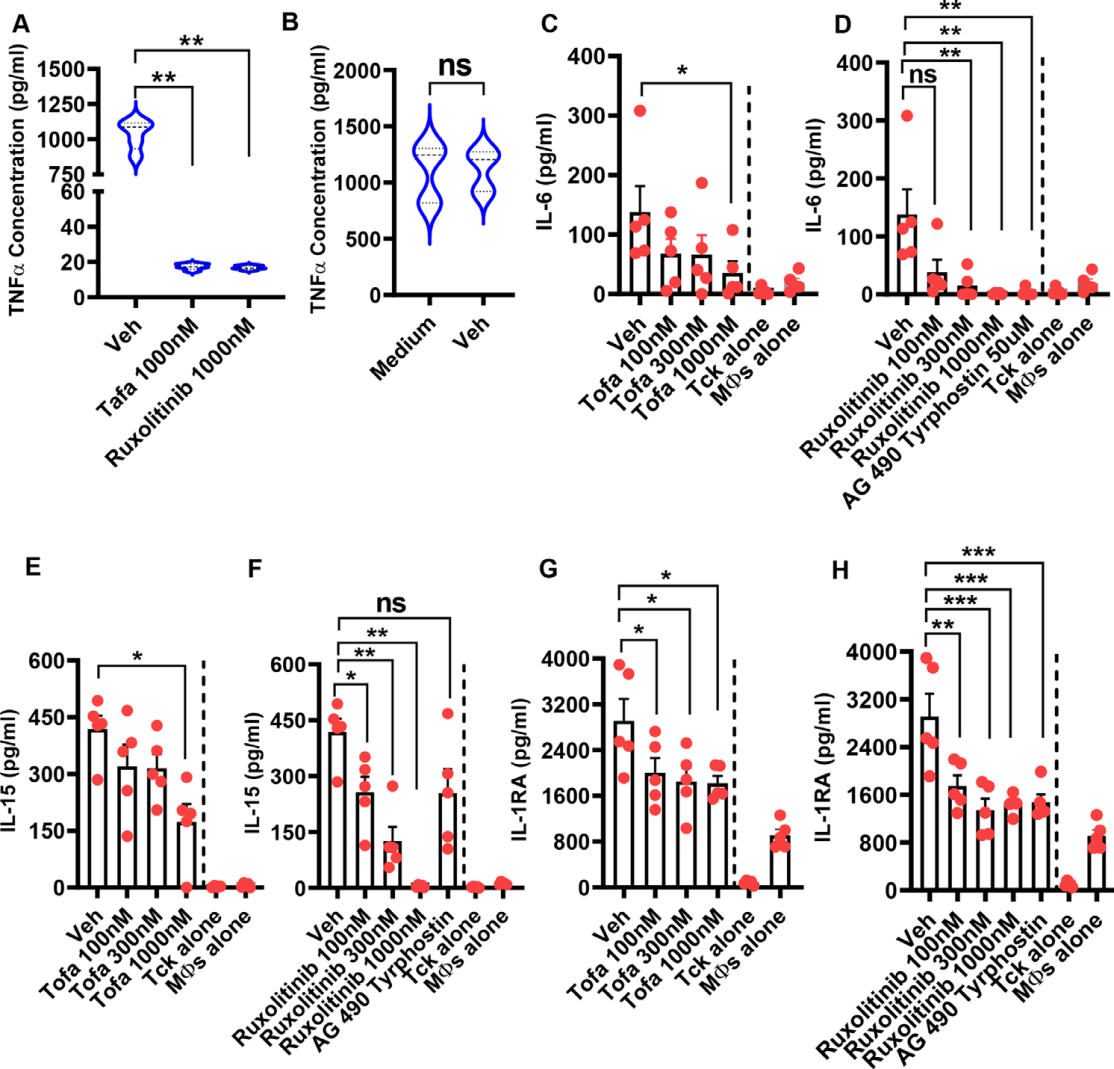
Janus kinase (JAK) inhibitors prevent cytokine-induced maturation of cytokine-activated T (Tck) cells and inhibit the production of IL-6, IL-15 and IL-1RA by activated macrophages. CD14^+^ monocytes (5×10^5^ cells/mL) were differentiated into macrophages by culturing them in the presence of macrophage-colony stimulating factor (MCSF; 50 ng/mL) for 6 days in a complete medium in a 96-well plate. Purified CD4^+^ T cells (1×10^6^ cells/mL) were stimulated with a cocktail of IL-2, IL-6 and TNF in the absence or presence of 0.001% DMSO (used as Vehicle), tofacitinib (1000 nM) or ruxolitinib (1000 nM). The cells were incubated for 6 days to generate Tck. The Tck populations were washed and then cocultured with monocyte-derived macrophages at a concentration of 4:1 Tck-macrophage ratio. The supernatants were harvested, and the level of TNF was measured by ELISA. (A) The comparison of TNF production between DMSO and Tofacitinib (Tofa)-treated or ruxolitinib-treated Tck. (B) The comparison of TNF production between medium and DMSO-treated Tck. In separate experiments, cytokine-generated Tck were cocultured with monocyte-derived macrophages (MФs) at 4:1 ratio in the absence or presence of 0.001% DMSO (Veh) or tofacitinib (100 nM, 300 nM and 1000 nM) or ruxolitinib (100 nM, 300 nM, 1000 nM, 50 µM). Culture supernatants were harvested, and the levels of (C, D) IL-6, (E, F) IL-15 and (G, H) IL-1RA were measured using Luminex. Data represent a mean of triplicate cultures±SE of the mean of at least five experiments. Statistically significant differences are indicated (*p< 0.05; **p<0.01; ***p<0.001). DMSO, dimethyl sulfoxide; ns, not significant; Veh, vehicle.

The effect of tofacitinib or ruxolitinib was evaluated on Tck cell maturation. JAK inhibitors diminished the capacity to induce TNF production by macrophages in coculture, suggesting that Tck cells failed to mature sufficiently to be able to activate macrophages (figure 1A). The reduced TNF production was not due to cell death caused by the toxicity of DMSO (figure 1B). Tofacitinib was selected as it is in clinical use, while ruxolitinib and AG-490 tyrphostin extended the generability of the findings. We chose a range of concentrations (online supplemental figure 2A, B) corresponding to approximate blood levels reflected by peak and trough dosage of tofacitinib by mouth.^14^ We also explored the effect of JAK inhibitors on IL-6, IL-15 and IL-1RA synthesis, relevant cytokines in inflammatory processes in RA. Tofacitinib, ruxolitinib and AG-490 tyrphostin reduced IL-6, IL-15 and IL-1RA (figure 1C–H).

### JAK inhibitors reduce MIP1α, MIP1β, MIG and IP10 production by TcK-activated macrophages

**Figure 2:**
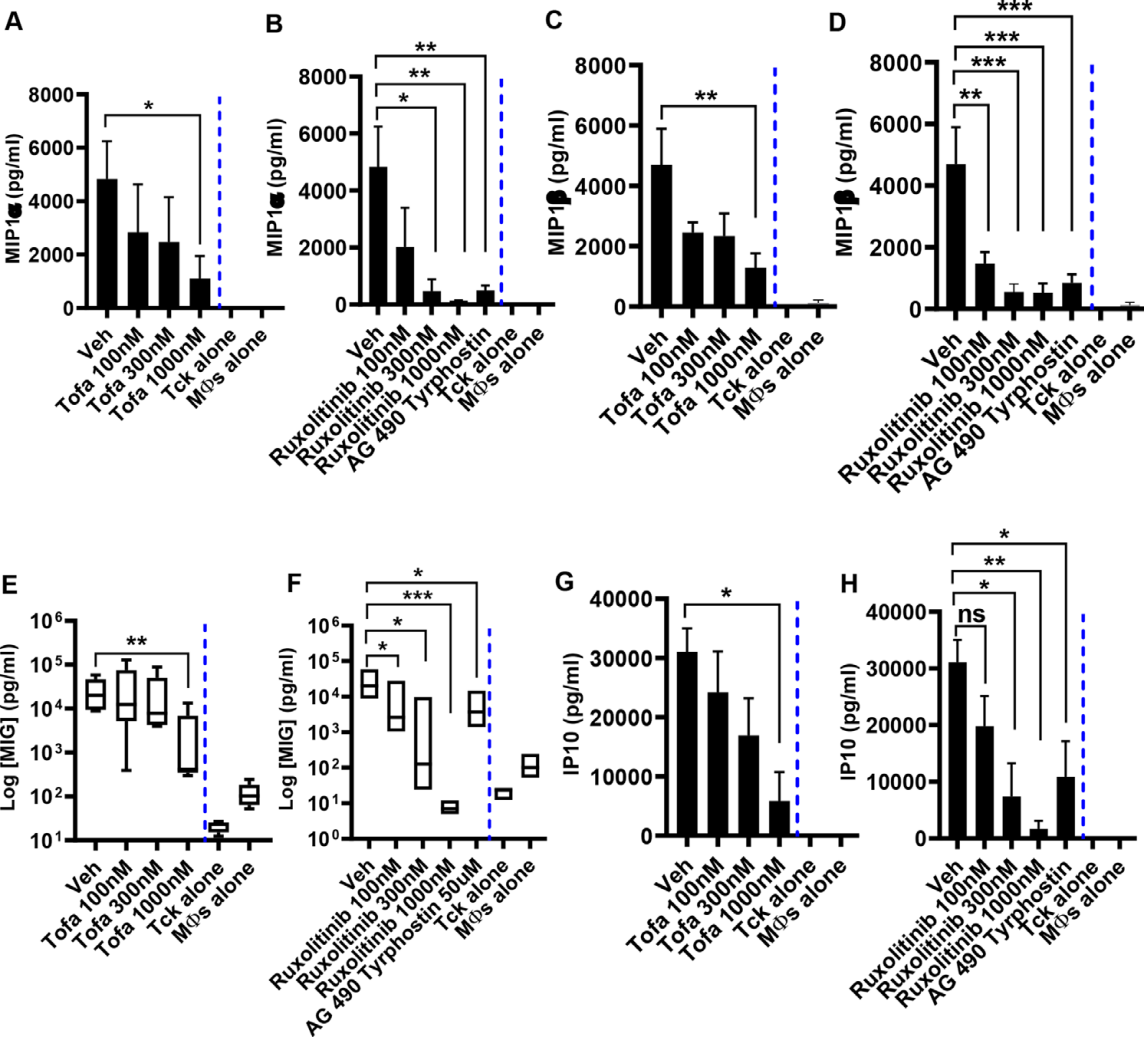
Janus kinase (JAK) inhibitors reduce MIP1α, MIP1β, MIG and IP10 production by activated macrophages. Cytokine-generated cytokine-activated T (Tck) cells were washed and then cocultured with monocyte-derived macrophages (MФs) at a 4:1 ratio. The cells were treated with 0.001% dimethyl sulfoxide (DMSO) (Veh) or tofacitinib (100 nM, 300 nM and 1000 nM) or ruxolitinib (100 nM, 300 nM, 1000 nM, 50 µM). In some experiments, MФs were also cultured alone. Culture supernatants were harvested, and the levels of (A, B) MIP1α; (C, D) MIP1β; (E, F) MIG; and (G, H) IP10 were measured using Luminex. Data represent a mean of triplicate cultures±SE of the mean of at least five experiments. Statistically significant differences are indicated (*p<0.05; **p<0.01; ***p <0.001; ns=not significant). Veh, vehicle.

We then evaluated the effect of JAK inhibitors on chemokines, including macrophage inflammatory protein MIP1α and MIP1β, monokine induced by interferon-γ (MIG)⁄CXCL9) and interferon-γ-inducible protein-10 (IP10)/CXCL10. These chemokines induce the synthesis and release of other proinflammatory cytokines that play pathological roles. At rest, macrophages or Tck cells did not produce MIP1α, MIP1β, MIG and IP10 (data not shown). Activation of macrophages with Tck cells induced increased production of MIP1α, MIP1β and MIG. The output of IP10 was generated by the treatment of macrophages with LPS. Treatment with tofacitinib, ruxolitinib or AG 490 tyrphostin reduced the release of MIP1α, MIP1β, MIG and IP10 (figure 2A–H).

## Discussion

We show that tofacitinib and ruxolitinib, two structurally unrelated small molecule inhibitors, inhibit the production of TNF, IL-6, IL-15, IL-1RA and chemokines MIP1α, MIP1β, MIG and IP10 by macrophages interacting with cytokine activated memory T-cells. This was due to tofacitinib and ruxolitinib preventing cytokine-induced maturation of Tck cells. Thus, tofacitinib and ruxolitinib impact the production of proinflammatory cytokines and chemokines crucial in the pathogenesis of immune-mediated inflammatory diseases. Although the effects of JAK inhibition on macrophages and T-cells have been studied,^15–18^ no study has thus far demonstrated the impact of JAK inhibition on Tck cell-activated macrophages.

Our observations concur with studies showing that JAK inhibitors mediate the suppression of TNF and IL-6 release, in keeping with the suggestion that JAK inhibitors work, in part, by suppressing the TNF-IFNβ-JAK-STAT1 autocrine loop.^17^ Tofacitinib, ruxolitinib and AG490 Tyrphostin also led to the inhibition of Tck cell maturation. Likely, the inhibition of IL-6 and IL-15 by tofacitinib and ruxolitinib contributed to the failure to generate mature Tck cells since these cytokines are crucial in forming mature Tck cells, as previously suggested.^12 13^ Indeed, we previously demonstrated that IL-15 (which signals via JAK1 and JAK3) is sufficient to form Tck cells and alters the balance between effector and memory T cells.^13^ These observations suggest that JAK inhibition may effectively disrupt cell contact-mediated inflammation at the synovial site and prevent a priori development of Tck cells.

Since Tck cells in our setting were not fixed, it is likely that a small proportion of the cytokines came from Tck cells and not all from macrophages. However, using fixed Tck cells, a previous study demonstrated that Tck cell-activated macrophages induced TNF^19^; therefore, it is reasonable to suggest that the TNF synthesis seen in our study is of macrophage origin.

The limitation of this study is that the investigation did not include RA patient samples, which would provide direct clinical relevance. However, the data suggest that JAK inhibition may effectively treat established inflammation by disrupting the crosstalk between macrophages and T-cells (online supplemental figure 3). Overall, JAK inhibition may affect multiple steps in mediating inflammation by targeting cytokine and chemokine production and affecting the function of innate and acquired immune cells, contributing to the beneficial effect of JAK inhibitors in inflammatory diseases like RA.

## References

[1] Croft AP, Campos J, Jansen K, et al. Distinct fibroblast subsets drive inflammation and damage in arthritis. Nature 2019;570:246–51. 10.1038/s41586-019-1263-7 31142839 PMC6690841

[2] Alivernini S, MacDonald L, Elmesmari A, et al. Distinct synovial tissue macrophage subsets regulate inflammation and remission in rheumatoid arthritis. Nat Med 2020;26:1295–306. 10.1038/s41591-020-0939-8 32601335

[3] Van Raemdonck K, Umar S, Palasiewicz K, et al. CCL21/CCR7 signaling in macrophages promotes joint inflammation and TH17-mediated osteoclast formation in rheumatoid arthritis. Cell Mol Life Sci 2020;77:1387–99. 10.1007/s00018-019-03235-w 31342120 PMC10040247

[4] Udalova IA, Mantovani A, Feldmann M. Macrophage heterogeneity in the context of rheumatoid arthritis. Nat Rev Rheumatol 2016;12:472–85. 10.1038/nrrheum.2016.91 27383913

[5] McInnes IB, Leung BP, Liew FY. Cell-Cell interactions in synovitis. interactions between T lymphocytes and synovial cells. Arthritis Res 2000;2:374–8. 10.1186/ar115 11094451 PMC130139

[6] Brennan F, Foey A. Cytokine regulation in RA synovial tissue: role of T cell/macrophage contact-dependent interactions. Arthritis Res 2002;4 Suppl 3:S177–82. 10.1186/ar556 12110137 PMC3240132

[7] Brennan FM, McInnes IB. Evidence that cytokines play a role in rheumatoid arthritis. J Clin Invest 2008;118:3537–45. 10.1172/JCI36389 18982160 PMC2575731

[8] Schwartz DM, Kanno Y, Villarino A, et al. Jak inhibition as a therapeutic strategy for immune and inflammatory diseases. Nat Rev Drug Discov 2017;16:843–62. 10.1038/nrd.2017.201 29104284

[9] Gao W, McGarry T, Orr C, et al. Tofacitinib regulates synovial inflammation in psoriatic arthritis, inhibiting STAT activation and induction of negative feedback inhibitors. Ann Rheum Dis 2016;75:311–5. 10.1136/annrheumdis-2014-207201 26353790 PMC4717390

[10] Fragoulis GE, McInnes IB, Siebert S. JAK-inhibitors. new players in the field of immune-mediated diseases, beyond rheumatoid arthritis. Rheumatology 2019;58:i43–54. 3. 10.1093/rheumatology/key276 30806709 PMC6390879

[11] McInnes IB, Byers NL, Higgs RE, et al. Comparison of baricitinib, upadacitinib, and tofacitinib mediated regulation of cytokine signaling in human leukocyte subpopulations. Arthritis Res Ther 2019;21:183. 10.1186/s13075-019-1964-1 31375130 PMC6679539

[12] Brennan FM, Hayes AL, Ciesielski CJ, et al. Evidence that rheumatoid arthritis synovial T cells are similar to cytokine-activated T cells: involvement of phosphatidylinositol 3-kinase and nuclear factor kappaB pathways in tumor necrosis factor alpha production in rheumatoid arthritis. Arthritis Rheum 2002;46:31–41. 10.1002/1529-0131(200201)46:1&lt;31::AID-ART10029&gt;3.0.CO;2-5 11822409

[13] McInnes IB, Leung BP, Sturrock RD, et al. Interleukin-15 mediates T cell-dependent regulation of tumor necrosis factor-alpha production in rheumatoid arthritis. Nat Med 1997;3:189–95. 10.1038/nm0297-189 9018238

[14] Cohen S, Zwillich SH, Chow V, et al. Co-administration of the JAK inhibitor CP-690,550 and methotrexate is well tolerated in patients with rheumatoid arthritis without need for dose adjustment. Br J Clin Pharmacol 2010;69:143–51. 10.1111/j.1365-2125.2009.03570.x 20233177 PMC2824475

[15] Ghoreschi K, Jesson MI, Li X, et al. Modulation of innate and adaptive immune responses by tofacitinib (CP-690,550). J Immunol 2011;186:4234–43. 10.4049/jimmunol.1003668 21383241 PMC3108067

[16] Kubo S, Yamaoka K, Kondo M, et al. The JAK inhibitor, tofacitinib, reduces the T cell stimulatory capacity of human monocyte-derived dendritic cells. Ann Rheum Dis 2014;73:2192–8. 10.1136/annrheumdis-2013-203756 24013646

[17] Yarilina A, Xu K, Chan C, et al. Regulation of inflammatory responses in tumor necrosis factor-activated and rheumatoid arthritis synovial macrophages by JAK inhibitors. Arthritis Rheum 2012;64:3856–66. 10.1002/art.37691 22941906 PMC3510320

[18] Maeshima K, Yamaoka K, Kubo S, et al. The JAK inhibitor tofacitinib regulates synovitis through inhibition of interferon-γ and interleukin-17 production by human CD4+ T cells. Arthritis Rheum 2012;64:1790–8. 10.1002/art.34329 22147632

[19] Sebbag M, Parry SL, Brennan FM, et al. Cytokine stimulation of T lymphocytes regulates their capacity to induce monocyte production of tumor necrosis factor-alpha, but not interleukin-10: possible relevance to pathophysiology of rheumatoid arthritis. Eur J Immunol 1997;27:624–32. 10.1002/eji.1830270308 9079801

