# Supplementary material for: JAK inhibitors disrupt T cell-induced proinflammatory macrophage activation
Source: RMD Open. 2023 Jan 4;9(1):e002671. doi: 10.1136/rmdopen-2022-002671 (PMC9815080; doi:10.1136/rmdopen-2022-002671)

Supplemental Material

Nyirenda et al

Supplemental Figure 1. Cytokine activated CD4+ T cells (Tck) induce contact- and concentration-dependent TNFα production by human macrophages.

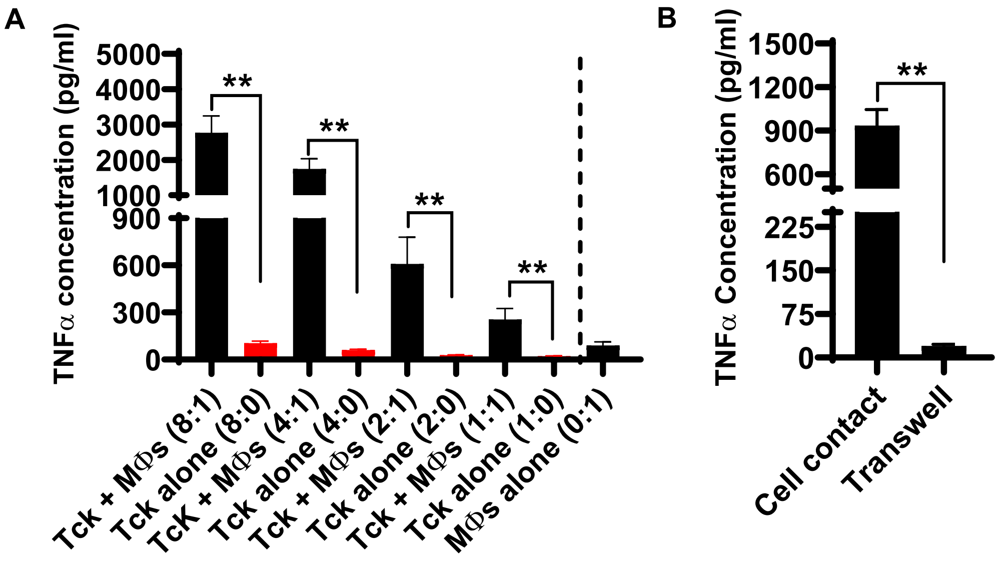

Supplemental Figure 2. Tofacitinib, ruxolitinib and AG-490 tryphostin inhibit TNFα production by Tck cell-activated macrophages.

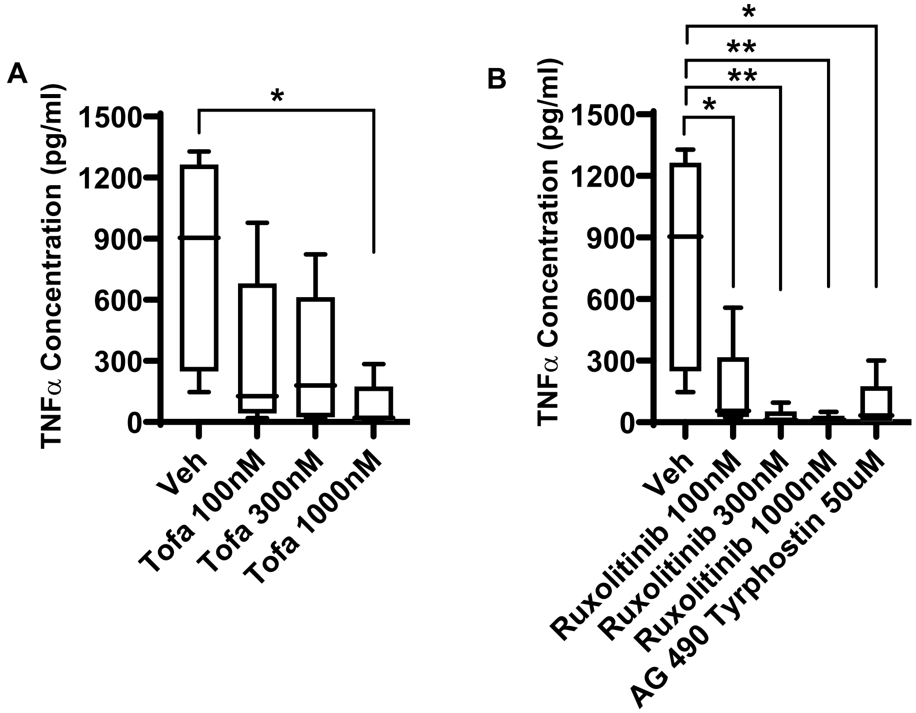

Supplemental Figure 6. JAK inhibition breaks the cytokine and chemokine inflammation cycle induced by cytokine-stimulated T cells (Tck).

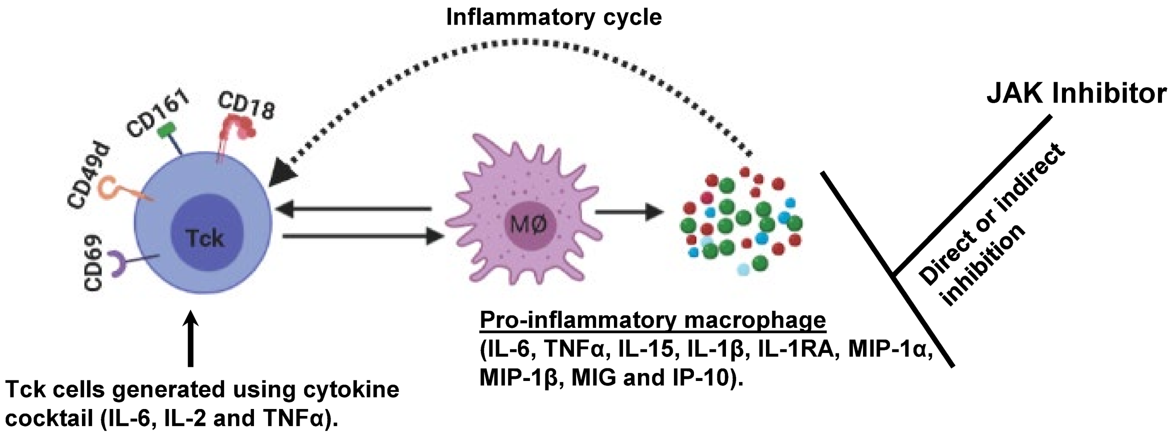

Supplement: Supplementary data [file rmdopen-2022-002671supp001.pdf]
